# Rotavirus A infection in pre- and post-vaccine period: Risk factors, genotypes distribution by vaccination status and age of children in Nampula Province, Northern Mozambique (2015-2019)

**DOI:** 10.1371/journal.pone.0255720

**Published:** 2021-08-06

**Authors:** Assucênio Chissaque, Adilson Fernando Loforte Bauhofer, Idalécia Cossa-Moiane, Ezequias Sitoe, Benilde Munlela, Eva Dora João, Jerónimo S. Langa, Jorfélia José Chilaúle, Simone Salvador Boene, Marta Cassocera, Esperança Lourenço Guimarães, Timothy A. Kellogg, Luzia Gonçalves, Nilsa de Deus

**Affiliations:** 1 Instituto Nacional de Saúde, Marracuene district, EN1, Maputo, Mozambique; 2 Instituto de Higiene e Medicina Tropical (IHMT), Universidade Nova de Lisboa, Lisboa, Portugal; 3 Institute of Tropical Medicine (ITM), Antwerp, Belgium; 4 Hospital Central de Nampula, Ministério de Saúde, Nampula, Mozambique; 5 Centro de Biotecnologia, Universidade Eduardo Mondlane, Maputo, Mozambique; 6 Institute for Global Health Sciences, University of California San Francisco, San Francisco, California, United States of America; 7 Global Health and Tropical Medicine and Unidade de Saúde Pública Internacional e Bioestatística, Instituto de Higiene e Medicina Tropical, Universidade Nova de Lisboa, UNL, Lisboa, Portugal; 8 Centro de Estatística e Aplicações da Universidade de Lisboa, Lisboa, Portugal; 9 Departamento de Ciências Biológicas, Universidade Eduardo Mondlane, Maputo, Mozambique; Federal University of Sergipe, BRAZIL

## Abstract

Mozambique introduced the monovalent rotavirus vaccine (Rotarix^®^, GSK Biologicals, Rixensart, Belgium) in September 2015. Previous analysis, showed that Nampula province continues reporting a high frequency of Rotavirus A (RVA) infection and the emergence of G9P[6], G9P[4] and G3P[4] genotypes. This analysis aimed to determine the RVA frequency; risk factors; genotype distribution by vaccination status and age between pre- and post-vaccine periods in children under-five years old with diarrhea in Nampula. A cross-sectional, hospital-based surveillance study was conducted in the *Hospital Central de Nampula* in Mozambique. Socio-demographic and clinical data were collected to assess factors related to RVA infection in both periods. Stool specimens were screened to detect RVA by ELISA, and positive samples were genotyped. Between 2015 (pre-vaccine period) and 2016–2019 (post-vaccine period), 614 stool specimens were collected and tested for RVA in which 34.9% (67/192) were positive in pre-vaccine period and 21.8% (92/422) in post-vaccine (p = 0.001). In the post-vaccine period, age, year, and contact with different animal species (chicken, duck, or multiple animals) were associated with RVA infection. RVA infection was higher in children partially vaccinated (40.7%, 11/27) followed by the fully vaccinated (29.3%, 56/191) and the unvaccinated (15.3%, 21/137) (p = 0.002). G1P[8] and G9P[4] were common in vaccinated children less than 12 months. The present analysis showed that RVA infection reduced slightly in the post-vaccine period, with a high proportion of infection and genotype diversity in children, under 12 months of age, vaccinated. Further research on factors associated with RVA infection on vaccinated compared to unvaccinated children and vaccination optimization should be done.

## Introduction

Globally, Rotavirus A (RVA) remains the leading cause of severe acute gastroenteritis associated with high childhood hospitalization and mortality, accounting for an estimated 128,515 deaths among children under five years old in 2016 [1, 2]. In Sub-Saharan Africa, RVA associated morbidity and mortality is exceptionally high with approximately 104,733 children dying annually from the disease [3]. In 2009, the World Health Organization (WHO) recommended the introduction of RVA vaccine in all countries, particularly in those with high child mortality [4]. In 2021, four attenuated oral rotavirus vaccines are licensed and available globally: Rotarix^®^ (GlaxoSmithKline Biologics, Rixensart, Belgium), RotaTeq^®^ (Merck & Co., USA), Rotavac^®^ (Bharat Biotech, India), and Rotasiil^®^ (Serum Institute of India Pvt. Ltd. India) [5]. A systematic review of the RVA vaccination impact on hospitalizations and deaths from 27 countries, found a 67% reduction in children’s hospitalization and outpatient care and a 60% decline in childhood mortality due to RVA gastroenteritis in 2016 [6]. Rotavirus morbidity decreased from 38% to 23% in 82 countries between 2008 and 2016 [7].

Studies of molecular characterization of RVA have shown that globally G1P[8] is the most common genotype combination, followed by G2P[4], G3P[8], G4P[8], and G9P[8] [8–11]. Following the introduction of the RVA vaccine (Rotarix^®^ and Rotateq^®^), new RVA strains were reported in Southern and Eastern African countries (Angola, Eritrea, Ethiopia, Kenya, Lesotho, Madagascar, Mauritius, Namibia, Rwanda, Seychelles, Swaziland, Tanzania, Uganda, Zambia and Zimbabwe) between 2010 and 2015; these included: G1P[4], G2P[8], G9P[4], and G12P[4] [11]. Before the RVA vaccine introduction in Mozambique, RVA was identified as the principal etiologic agent of moderate-severe-diarrhea with an attributable fraction of 34.8% in children under one year in the southern region of Mozambique [12]. Other studies in children with diarrhea conducted in the southern region (Chókwè, Maputo city, and Manhiça) showed RVA frequencies ranging from 24% to 42.4% [13, 14]. Those studies reported mostly the following circulating genotypes: G12P[8] (57%) and G1P[8] (9%) in Chòkwé, G2P[4] (39.4%) in Manhiça and G12P[6] (28.6%) in Maputo city [14, 15]. Mozambique introduced the G1P[8] rotavirus vaccine (Rotarix^®^) into the expanded program of immunization in September 2015. Before Rotarix^®^ introduction, the *Instituto Nacional de Saúde* (INS) implemented the National Surveillance of Diarrhea (ViNaDia) in four provinces of the country as a platform to monitor diarrhea cases, the main etiologies, and risk factors associated with RVA infection before and after Rotarix^®^ introduction in the country. ViNaDia data indicated a high diversity of RVA genotypes across the country, were uncommon combinations such as G3P[4], G9P[4], G9P[6], G2P[6], and non-typeable genotypes in Nampula province were seen [16]. ViNaDia also reported a high proportion of undernourished children with the double burden of RVA and HIV [17]. An independent analysis of RVA in Nampula is relevant because recurrent reports of poorer health indicators in children are described, including a high burden of conditions such as undernourishment, malaria, diarrhea and successive cholera outbreaks [18, 19]. Thus, the present analysis aims to determine RVA frequency, potential risk factors and the distribution of RVA genotypes by vaccination status and age among children with diarrhea attending *Hospital Central de Nampula*, in the pre- and post-vaccine periods.

## Materials and methods

### Study design, site, population

A cross-sectional, hospital-based surveillance was conducted between the pre-vaccine (March—December 2015) and post-vaccine (January 2016 –December 2019) periods. The surveillance was conducted at the *Hospital Central de Nampula* (HCN) in Nampula province, located in the northern region of Mozambique. Nampula province is highly populated, and has enormous challenges related to Water, Sanitation and Hygiene (WASH), high levels of poverty, and lower vaccination coverage [19, 20]. Children aged up to 59 months who presented to the HCN pediatric services with diarrhea, defined as the passage of three or more loose or liquid stools in the last 24 hours before seeking healthcare, were surveyed [21]. Children were not included in this analysis if the caregivers did not consent to their participation as well as children diagnosed with nosocomial diarrhea. Moreover, children considered overweight (+2 < Z-score ≤ +5) or with outlier Z-scores values were excluded from the nutritional status analysis [22].

### Data collection

Data was collected by interviewing the child’s caregiver and by accessing the child’s medical records. The main variables include age (in months), categorized as 0–11, 12–23, 24–59; sex; exclusive breastfeeding; the age of weaning in months, categorized by six months; animal contact and type of animal; source of drinking water; HIV status; admission type; number of diarrhea episodes in the last 24h; vomit; fever; weight; vaccination status, and nutritional status that was estimated through the weight-for-age Z-score (WAZ), specific to underweight, using WHO software Anthro version 3.2.2. Weight was measured by lying down children younger than two years, and standing for children with two years or older. Children were classified as well-nourished if the Z-score was between -2 ≤ Z-score ≤ +2 and as underweighted if the z-scores was between -6 ≤ Z-score < -2. Vaccination status of the children was confirmed with a valid vaccination card. Trained healthcare professionals of ViNaDia, completed all information on a structured data collection form.

### Sample collection

ViNaDia methodology has been previously described elsewhere [16, 17, 23, 24]. Briefly, a single stool specimen (10 ml) was collected from each child in a polyester container and stored at -20°C until shipment, once a week, to the INS laboratory in Maputo for diagnosis.

### Laboratory procedures

Collected samples were tested for RVA using the commercial enzyme-immuno-sorbent assay (ELISA) kit (Prospect, Oxoid Ltd, United Kingdom) according to the manufacturer’s instructions. All positive samples were submitted to total RNA extraction using the QIAamp Viral RNA protocol (QIAGEN, Hilden, Germany) following the manufacturer’s instructions.

Extracted RNA (8μl) was reverse transcribed and amplified using a reverse-transcription polymerase chain reaction (RT-PCR) with Avian Myeloblastosis Virus (AMV), reverse transcriptase (Ivitrogen, USA), Taq DNA polymerase (Ivitrogen, USA) and the primers sBeg9/End9 for the VP7 encoding gene (1062pb) and Con2/Con3 for the partial VP4-encoding gene (VP8*, 876bp) were used [25, 26]. Amplicon products from the first round RT-PCR were added to a second-round multiplex semi-nested PCR containing RVG9 and specific primers for G-type (G1-4, G9-10, and G12) identification were used as described elsewhere [16, 25, 27, 28].

Similarly, Con3 was used in combination with specific primers that identify P-types (P[4], P[6], P[8], P[9], P[11], and P[14]) as previously described [16, 26, 29]. The cycling conditions consisted of 1 minute denaturation, followed by 30 cycles of dsRNA denaturation for 1 minute at 95°C, annealing for 1 minute at 42°C, 1 minute at 72°C for amplification, and the final amplification step of 7 minutes at 72°C.

The PCR product was analysed using 2% agarose gel electrophoresis, stained with ethidium bromide, and visualized under ultraviolet illumination.

### Sample size

Data used in the present analysis are from a hospital-based surveillance program (ViNaDia), whose primary aim is to monitor diarrhea and RVA cases before and after Rotarix^®^ vaccine introduction [17]. Since ViNaDia is an ongoing surveillance system, the sample size calculation was not estimated for the present analysis. However, we calculated the statistical power associated to the obtained sample sizes to compare two proportions. For children aged up to 59 months, statistical power was 92.1%. For the secondary analysis in children younger than 24 months, the sample power was 88.0% (higher than 80% minimum required).

### Data management and statistical analysis

Data collected was double entered in two similar databases by two independent typists in Epi Info^™^3.5.1. (Centers for Disease Control and Prevention, Atlanta, 2008), followed by data comparison. IBM SPSS (International Business Machines Corporation Statistical Package for the Social Science, Armok, NY: IBM Corp, 2011, version 26.0) software was used to conduct the data analysis.

Overall RVA frequencies with the corresponding Wilson’s 95% Confidence Interval (CI) were estimated, for each RVA vaccine period and year [30]. Also, RVA proportions by age (in months) groups for each year were calculated.

Descriptive statistics and chi-square test or Fisher’s Exact test were used to identify socio-demographic and epidemiological variables associated with RVA infection in each vaccine period in children age up to 59 months and in children younger than 24 months (S1 Table). Due to the absence of RVA cases among children aged between 24–59 months in the post-vaccine period, a more detailed analysis was done to children under 24 months. Multiple logistic regression models were explored to obtain adjusted odds ratio (aOR), with 95% confidence intervals, for each vaccine period. The bivariate analysis of the socio-demographic and epidemiological characteristics for children under 24 months of age is shown in S1 Table. Variables with initial p < 0.05 were included in the multiple logistic regression model for children under 24 months of age. Goodness-of-fit of the multiple logistic regression models was obtained using Hosmer and Lemeshow test.

For children aged up to 59 months, clinical factors associated with RVA infection in each vaccine period and in general were assessed by cross-tabulation followed by the chi-square test or Fisher’s Exact test if assumptions were not met. Cross-tabulation was performed between vaccination status and RVA infection; vaccination status and genotype distribution; age and genotype distribution. Parameter estimation was done using 95% confidence interval, and hypothesis test were based on a 5% level.

### Ethical statement

The study protocol was approved by the National Bioethics Committee for Health in Mozambique (IRB00002657, reference 348/CNBS/13). Parents or legal guardians of the children signed or provided their fingerprint to the informed consent form, which described the study objectives and clarified that participation in the study was voluntary. Data confidentiality was ensured by storing the physical data collection and consent forms in a lockable cabinet with limited access.

## Results

### Overall and yearly RVA frequencies by vaccination period

In total, 700 children were recruited by ViNaDia in Nampula hospital (221 from the pre-vaccine period and 479 from the post-vaccine period), of which 614 (87.7%) provided a stool sample for laboratory procedures, (192 from the pre-vaccine period and 422 from the post-vaccine period).

**Fig 1:**
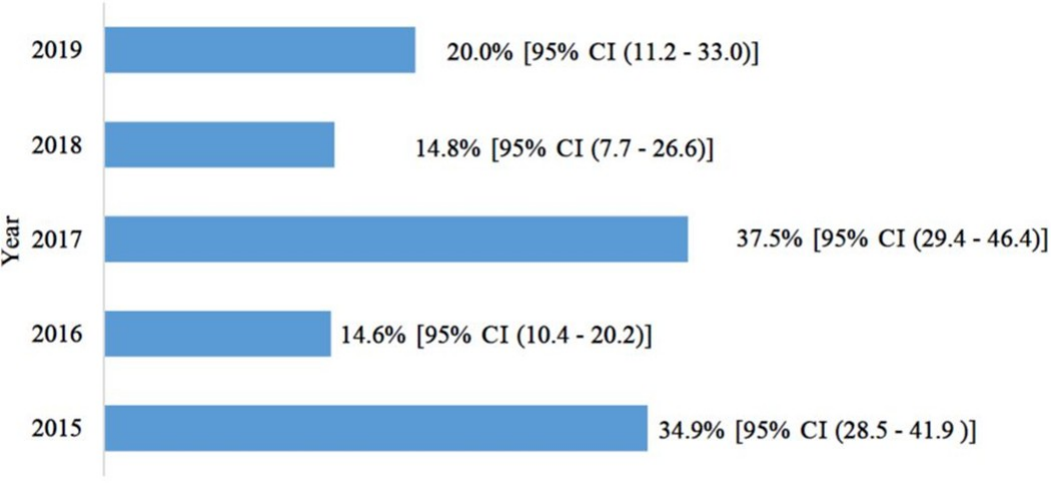
Frequency of RVA infection by year and their Wilson’s confidence intervals.

Overall, 25.9% (159/614) of the children were positive for RVA- 95% CI: 22.6–29.5. RVA infection was more frequent in the pre-vaccine (2015) period [34.9% (95% CI: 28.5–41.9); 67/192] than in the post-vaccine (2016–2019) period [21.8% (95% CI: 18.1–26.0); 92/422] (p = 0.001). RVA frequency by year after vaccine introduction was 14.6% (29/198) in 2016, 37.5% (45/120) in 2017, 14.8% (8/54) in 2018 and 20.0% (10/50) in 2019 (Fig 1).

### RVA frequency by age group and year

**Fig 2:**
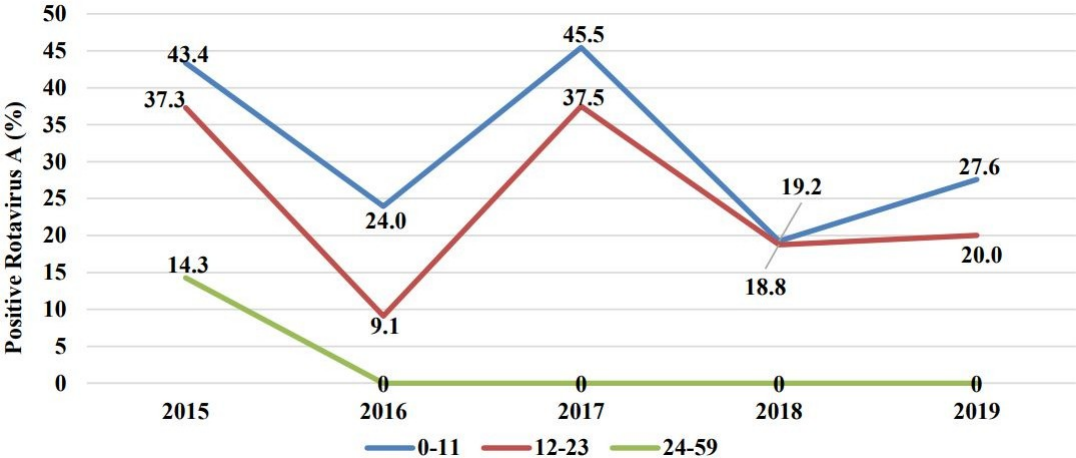
Frequency of RVA per year and children age groups in *Hospital Central de Nampula*.

RVA infection was more frequent among children under 12 months of age than the older ones in each analyzed year, 43.4% (36/83) in 2015, 24.0% (23/96) in 2016, 45.5% (30/66) in 2017, 19.2% (5/26) in 2018 and 27.6% (8/29) in 2019 (Fig 2). RVA infection in children between 24–59 months was only observed in the pre-vaccine period, 14.3% (6/42). Each year, the median age (in months) of positive children was below 12 months, in which the third quartile was lower than 24 months, being 11 (8–15) for 2015, 9 (7–11) for 2016, 10 (8–12.5) for 2017, 8.5 (7.25–12) for 2018 and 9.5 (8–11.25) for 2019.

### Potential risk factors for RVA infection in pre- and post-vaccine period in *Hospital Central de Nampula*

In the pre-vaccine period, RVA infection was more frequent in children younger than 12 months (p < 0.010) (Table 1). Exclusive breastfeeding (44.4%; 32/72; p < 0.028); drinking water from a piped source (54.7%; 35/64; p < 0.001) and Borehole/well (18.8%; 13/69; p < 0.001) were also associated with RVA infection in the pre-vaccine period. Underweight was negatively associated with RVA infection, as the infection was more frequent in well-nourished children (42.2%; 43/102; p < 0.003) (Table 1).

**Table 1:** Frequencies of RVA infection by socio-demographic and epidemiological characteristics in the pre- and post-vaccine period, Nampula, Mozambique.

Characteristics	Pre-vaccine n/N	%	p	Post-vaccine n/N	%	p
**Age categorized (in months)**			**0.005**			**<0.001**
0–11	36/83	43.4		66/217	30.4	
12–23	25/67	37.3		26/132	19.7	
24–59	6/42	14.3		0/73	0.0	
**Sex**			0.056			0.922
Male	30/104	28.8		56/255	22.0	
Female	37/88	42.0		36/167	21.6	
**Exclusive breastfeed**			**0.028**			0.947
Yes	32/72	44.4		14/63	22.2	
No	34/118	28.8		78/357	21.8	
**Animal contact**			0.843			**<0.001**
No	56/161	34.8		17/145	11.7	
Yes	11/30	36.7		75/277	27.1	
**Source of drinking water**						
**Public tap**			0.628			0.483
No	48/134	35.8		31/155	20.0	
Yes	17/53	32.1		61/266	22.9	
**Piped water**			**<0.001**			0.967
No	30/123	24.4		74/338	21.9	
Yes	35/64	54.7		18/83	21.7	
**Borehole/well**			**<0.001**			0.327
No	52/118	44.1		80/352	22.7	
Yes	13/69	18.8		12/69	17.4	
**River/lake/lagoon**			1.000 ^a^			1.000 ^a^
No	65/186	34.9		92/419	22.0	
Yes	0/1	0.0		0/2	0.0	
**Purchased/bottled water**			NA			0.219 ^a^
No	65/187	34.8		91/420	21.7	
Yes	0/0	0.0		1/1	100.0	
**Age weaning in months (categorized)**			0.579 ^a^			**0.004** ^a^
0–6	1/4	25.0		3/11	27.3	
7–12	2/20	10.0		3/18	16.7	
13–24	4/30	13.3		1/68	1.5	
25–59	1/5	20.0		0/4	0.0	
**HIV**			0.446			1.000 ^a^
Negative	54/163	33.1		30/183	16.4	
Positive	4/8	50.0		2/13	15.4	
**Underweight (weight-for-age)**			**0.003**			**0.044**
No	43/102	42.2		64/262	24.4	
Yes	12/62	19.4		23/145	15.9	

^a^ Fisher’s Exact Test.

NA: Not Applicable.

Similarly, to the pre-vaccine period, in the post-vaccine period, the child’s age and weight-for-age were also associated to RVA infection, p < 0.001 and p = 0.044 respectively. Several factors that were not associated with RVA infection in the pre-vaccine period were associated with RVA infection in the post-vaccine period. These included animal contact (27.1%; 75/277; p < 0.001) and weaning within the first six months of life (27.3%; 3/11; p = 0.004) (Table 1).

### Type of animal which children had contact in pre- and post-vaccine periods

The animal species that children had contact in pre- and post-vaccine periods show that none of the animals were associated with RVA infection in pre-vaccine period (Table 2). However, it was observed an association between chicken contact (37.2%; 29/78; p <0.001); duck (37.0%; 17/46; p = 0.008), or multiple animals (30.6%; 34/111; p = 0.009) contact with RVA infection in the post-vaccine period (Table 2).

**Table 2:** Type of animal which children had contact in the pre- and post-vaccine periods.

Characteristics	Pre-vaccine n/N	%	p	Post-vaccine n/N	%	p
**Animal species**						
**Pig**			0.351^a^			0.208^a^
No	66/190	34.7		90/418	21.5	
Yes	1/1	100.0		2/4	50.0	
**Goat**			NA			0.595^a^
No	67/191	35.1		86/400	21.5	
Yes	0/0	0.0		6/22	27.3	
**Mouse**			1.000^a^			0.226
No	66/189	34.9		67/327	20.5	
Yes	1/2	50.0		25/95	26.3	
**Dog**			0.659^a^			0.956
No	66/186	35.5		78/357	21.8	
Yes	1/5	20.0		14/65	21.5	
**Duck**			NA			**0.008**
No	67/191	35.1		75/376	19.9	
Yes	0/0	0.0		17/46	37.0	
**Chicken**			1.000^a^			**<0.001**
No	66/189	34.9		63/344	18.3	
Yes	1/2	50.0		29/78	37.2	
**Cat**			0.865			0.826
No	60/172	34.9		65/302	21.5	
Yes	7/19	36.8		27/120	22.5	
**Multiple animals**			1.000^a^			**0.009**
No	67/190	35.3		58/311	18.6	
Yes	0/1	0.0		34/111	30.6	

NA—Not applicable.

^a^ Fisher’s Exact Test.

In the multivariable analysis for the pre-vaccine period, children from household’s that drunk piped water had 2.49 times greater odds of being infected by RVA compared to the children who did not drink piped water (aOR = 2.49, 95% CI: 2.49 1.04–5.95; p < 0.040) (Table 3).

**Table 3:** The adjusted odds ratio for infection by RVA in pre- and post-vaccine periods, in children below 24 months.

Characteristics	Pre-vaccine		Post-vaccine	
	p	aOR (95% CI)	p	aOR (95% CI)
**Age (in months)**				
0–11	0.354	1.40 (0.69–2.86)	**0.028**	**1.83 (1.07–3.13)**
12–23		Ref		Ref
**Sex**				
Male		Ref		Ref
Female	0.097	1.83 (0.90–3.75)	0.873	1.04 (0.62–1.75)
**Year of admission**	NA		0.004	
2015			NA	NA
2016		NA	0.814	0.90 (0.38–2.15)
2017		NA	**0.038**	**2.40 (1.05–5.50)**
2018		NA	0.687	0.80 (0.28–2.34)
2019		NA		Ref
**Animal contact**				
No	NA	NA		Ref
Yes	NA	NA	**0.048**	**1.92 (1.01–3.66)**
**Source of drinking water**				
**Piped water**				
No		Ref	NA	NA
Yes	**0.040**	**2.49 (1.04–5.95)**		NA
**Borehole/well**				
No		Ref	NA	NA
Yes	0.140	0.51 (0.21–1.25)		NA

NA—Not applicable.

Ref—Reference category.

aOR—Adjusted Odds Ratio.

CI—Confidence Intervals.

In the post-vaccine period, children between 0–11 months of age were 1.83 times more likely of being infected by RVA than the children between 12–23 months (aOR = 1.83; 95% CI: 1.07–3.13; p = 0.028). After Rotarix^®^ introduction, year 2017, was the one with the highest chances of infection by RVA compared to 2019 (aOR = 2.40; 95% CI: 1.05–5.50; p = 0.038). Children with animal contact were 1.92 times more likely to be infected by RVA compared to children without animal contact (aOR = 1.92; 95% CI: 1.01–3.66; p = 0.048) (Table 3).

### Clinical factors related to RVA infection in children from *Hospital Central de Nampula*

Overall, the factors related to RVA infection were a greater number of diarrhea episodes in the last 24h before admission (p = 0.041); and the occurrence of vomiting (30.6%; 126/418; p < 0.001) (Table 4). In the pre-vaccine period occurrence of fever was the only clinical factor related to RVA infection (48.4%; 15/31; p = 0.038) (Table 4).

**Table 4:** Clinical factors related to infection by RVA in general and by vaccine period.

Characteristics	Overall	p	Pre-vaccine	p	Post-vaccine	p
	% (n/N)		% (n/N)		% (n/N)	
**Admission type**		0.924		0.315 ^a^		0.324
Inpatient	26.0 (143/551)		35.4 (67/189)		21.0 (76/362)	
Outpatient	25.4 (16/63)		0.0 (0/3)		26.7 (16/60)	
**Diarrhea episodes in the last 24h**		**0.041**		0.625		**0.008**
1–3	18.1 (23/127)		31.4 (16/51)		9.2 (7/76)	
4–5	29.8 (89/299)		39.7 (31/78)		26.2 (58/221)	
≥6	25.0 (36/144)		37.5 (9/24)		22.5 (27/120)	
**Vomiting**		**<0.001**		0.270		**0.001**
No	16.1 (31/193)		27.5 (11/40)		13.1 (20/153)	
Yes	30.6 (128/418)		36.8 (56/152)		27.1 (72/266)	
**Fever**		0.436		**0.038**		0.908
No	25.9 (90/347)		28.6 (32/112)		24.7 (58/235)	
Yes	29.4 (42/143)		48.4 (15/31)		24.1 (27/112)	

^a^ Fisher’s Exact Test.

In the post-vaccine period, clinical factors related to infection by RVA, were the number of diarrhea episodes in the last 24h, infection was more common in children with four or more episodes (p < 0.01); and occurrence of vomit (27.1%, 72/266; p = 0.001) (Table 4).

### RVA infection by vaccination status of the children in *Hospital Central de Nampula*

A stratified analysis between RVA infection and vaccination status was performed for the post-vaccine period, and RVA infection was more common in partially vaccinated children (40.7%, 11/27) followed by the fully vaccinated (29.3%, 56/191) and unvaccinated (15.3%, 21/137) (p = 0.002).

The most common strains identified were G9P[6] (33.3%; 4/21) in the unvaccinated children, G9P[4] (45.5%; 5/11) in the partially vaccinated, and G1P[8] (30.4%; 17/56) in the fully vaccinated. Also, most diversity was found in the fully vaccinated children, including the uncommon combinations such as G3P[4] with 10.7% (6/56) and G9P[4] with 17.9% (10/56). Non-typeable samples were also found in fully vaccinated children. The mixed infection (G1G3P[8]) were most common in the partially vaccinated group, with 18.2% (2/11) (Table 5).

**Table 5:** RVA genotypes combination identified by vaccination status in the post-vaccination period.

Genotypes	Vaccination status					
	Unvaccinated	%	Vaccinated (first dose)	%	Vaccinated (full doses)	%
G1P[8]	4	19.0	1	9.1	**17**	**30.4**
G2P[6]	1	4.8	0	0.0	3	5.4
G3P[4]	0	0.0	0	0.0	6	10.7
G3P[8]	0	0.0	0	0.0	2	3.6
G9P[4]	5	23.8	**5**	**45.5**	10	17.9
G9P[6]	**7**	**33.3**	1	9.1	8	14.3
G9P[8]	0	0.0	1	9.1	0	0.0
G12P[8]	0	0.0	1	9.1	0	0.0
G1G3P[8]	1	4.8	2	18.2	1	1.8
G9P[X] ^a^	0	0.0	0	0.0	1	1.8
GX^b^P[4]	0	0.0	0	0.0	5	8.9
GX^b^P[6]	2	9.5	0	0.0	0	0.0
GX^b^P[8]	0	0.0	0	0.0	1	1.8
GX^b^P[X] ^a^	1	4.8	0	0.0	2	3.6
Total	21	100.0	11	100.0	56	100.0

^a^ Refers to strains that were non-typeable for P.

^b^ Refers to strains that were non-typeable for G.

### RVA genotypes distribution by age group in pre- and post-vaccine period in *Hospital Central de Nampula*

**Fig 3:**
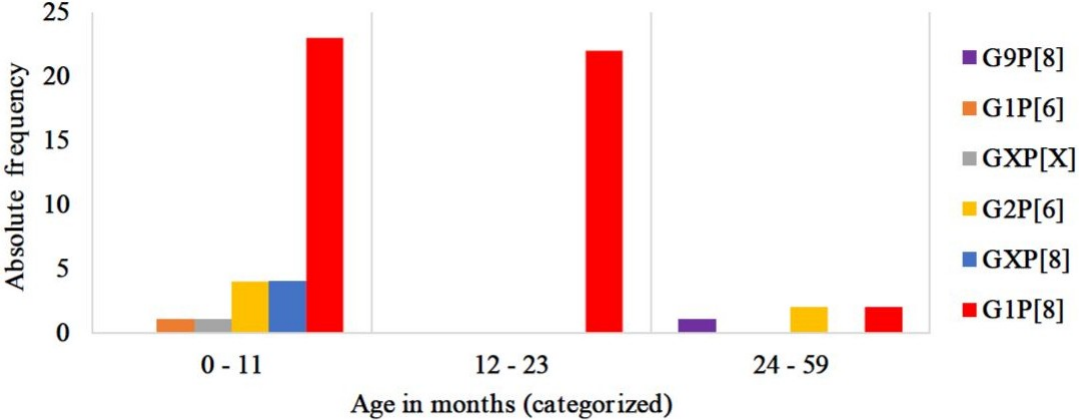
RVA genotypes distribution by age groups in the pre-vaccine period; N = 60.

Sixty out of sixty-seven (89.6%) RVA positive samples from the pre-vaccine period had a sufficient amount for RT-PCR. G1P[8] genotype combination was the most common among children younger than 24 months of age (45/55) (Fig 3., S2 Table).

**Fig 4:**
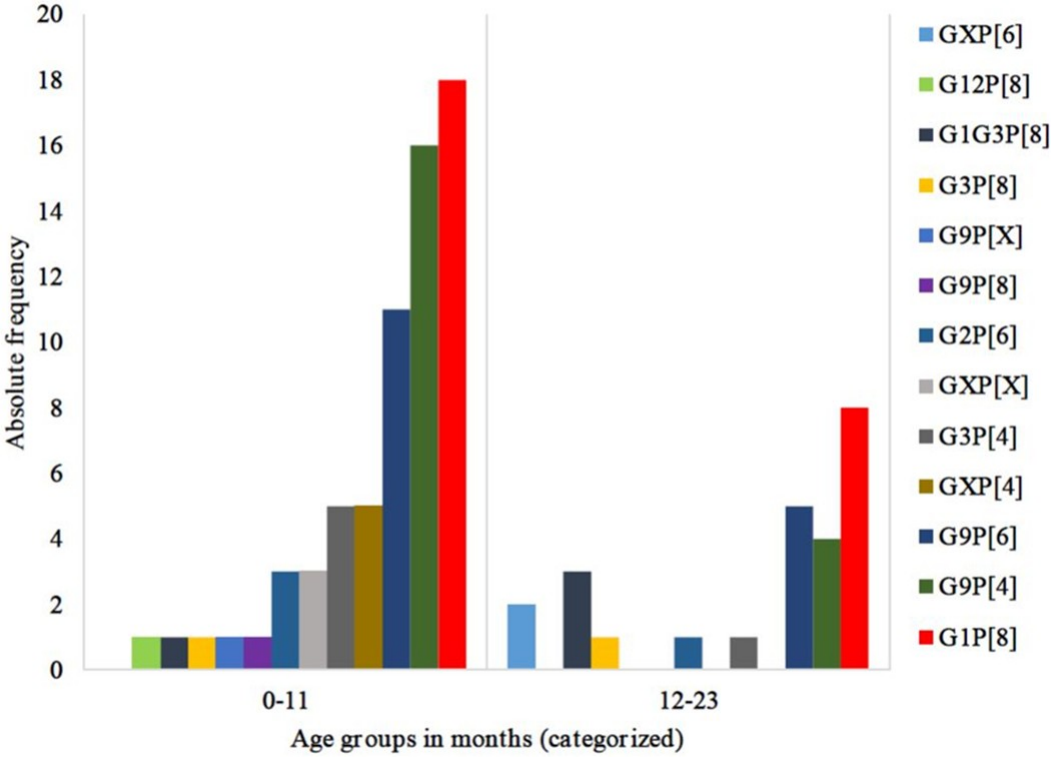
Genotypes distribution by age groups in the post-vaccine period; N = 92.

All RVA positive samples collected in the post-vaccine period were submitted to molecular characterization and were from children below 24 months of age. The G1P[8] was the most common with 28.3% (26/92). However, in children younger than 12 months of age, a higher frequency of emergent strains that were not detected in the pre-vaccine period were observed, including mixed infections. In the older group (12–23 months) G1P[8] 100% (22/22) was the only genotype reported in the pre-vaccine period (Fig 3). In contrast, in the post-vaccine period, other strains, such as G9P[6] (19.2%, 5/26) and G9P[4] (15.4%, 4/26), were also reported (Fig 4, S3 Table).

### Monthly distribution of RVA infection in pre- and post-vaccine period

**Fig 5:**
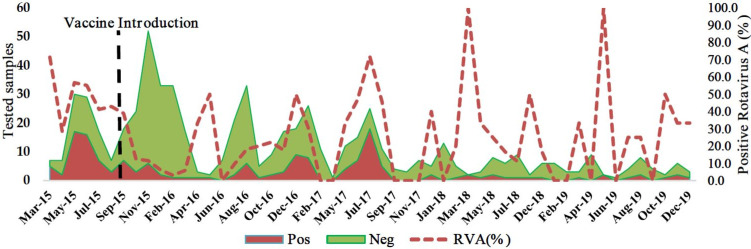
Monthly distribution of RVA infection between March 2015 and December 2019.

Since the introduction of the vaccine in the country, the number of diarrhea and RVA cases have declined, mostly since late 2017 at *Hospital Central de Nampula*. RVA cases occurred in both seasons, cold/dry and wet/rainy. The frequency of RVA during the cold/dry season (April to September) was 35.8% (108/302) and in the wet/rainy season (October to March) was 16.3% (51/312) (p < 0.001) (Fig 5).

## Discussion

We investigated the epidemiology of RVA, and the distribution of genotypes by vaccination status and age among children with diarrhea attending *Hospital Central de Nampula*, in Nampula province northern region of the country. Our analysis showed that RVA infection declined following vaccine introduction by 13.1%. In comparison, two other studies in Maputo city, a southern region of the country, found a drop in RVA infection of 31.2% and 14.2% respectively [23, 31]. The differences observed can be explained by the lower vaccine coverage in Nampula province (46%) compared to Maputo city (61%) (data not published from the Expanded Program on Immunization) in 2016, and the lower health indicators in Nampula province [32].

Despite the reduction in RVA morbidity during the post-vaccine period, an increase in RVA infection frequency was seen in 2017. This event may be explained by the lower RVA vaccine coverage [33], and the impact of increasing the surveillance after introducing of RVA vaccine in the country.

After the vaccine introduction, there was a reduction of RVA infection in different age groups, with a significant decrease among children from 24–59 months of age. However, the frequency of RVA infection remains high in children under 12 months of age. This finding differs from an analysis from *Hospital Geral de Mavalane* and *Hospital Central de Maputo*, which reported a substantial reduction of RVA infections among children under 12 months of age, with a shift to older age-groups [14, 23].

Differences in the age infection shifting may be due to lower vaccination coverage in Nampula [32], reinforced by our data in which 38.6% of the children were not vaccinated even being eligible for vaccination in post-vaccine period.

RVA is a zoonotic agent with a risk of interspecies transmission [34, 35]. In Chókwè, a rural area of Mozambique, phylogenetic data of G4P[6] strain detected in children, clustered with porcine and human porcine-like strains [14]. Animal contact with duck, chicken or multiple animals points to a potential association of RVA interspecies transmission. The occurrence of RVA in chickens was reported in Nigeria [36] and Kenya [37]. Furthermore, a study conducted in Germany identified an avian VP4 gene, strain P [37] closely related to mammalian RVA [38]. These data highlight the need of expanding the RVA surveillance to domestic animals in Mozambique.

Mozambique has problems related to the availability of water and as a strategy to mitigate the scarcest; intermittently distribution of water between few hours during the day was adopted, which forces households to storage the water in recipients for long use. Our surveillance has limited information on the conditions that the piped water were stored, or in what extent would be safe for consumption after storage. Most of the Mozambican pipelines are old and damaged in some sections aligned with the fact that are not properly maintained [39, 40], which can contribute for contamination of the piped water mainly in flood situation as the supply network are emerged [39, 41, 42]. A recent study conducted in the capital of the country (Maputo), which analyzed different sources of water (home bottled, piped and supply well water) observed higher microbiological contamination than recommended for human consumption in piped water [42]. Although the study was carried out in Maputo, the company that supplies water at the national level is the same. If so, this finding helps to explain the high frequency of RVA in children who consume piped water in Nampula.

The Demographic and Health Survey showed that only 43% of the children under six months are solely breastfed in Mozambique [19]. Surprisingly in this analysis, RVA infection was more common in children exclusively breastfeed. Similar results were reported in Kenya in children under 24 months, where a high proportion of positive children were exclusively breastfed than those with other types of food, with 16% and 10.3% respectively in the post-vaccine period [43]. One of the hypotheses behind this finding is related to the maternal antibodies which may interfere with the RVA vaccine efficacy in lower and middle-income countries, however, the causal link is not well described [44].

The fact that the proportion of RVA infected children with fever, vomiting, multiple diarrhea episodes, or visit requiring hospitalizations decreased in the post-vaccine period, may highlight the role of vaccination in reducing the severity of RVA infection [45, 46].

More than one-third of the children who received only the first dose of vaccine had a high frequency of RVA infection, which raises the question of whether they are shedding the vaccine strain. Vaccine shedding was accessed and the minimum days between the symptoms onset and the vaccination day for dose one were 32 days and for dose two were 26 days (data not shown), which excludes the possibility of shedding [47]. This result indicates a need for further research in Nampula to understand which factors are behind the high frequency of infection in vaccinated children.

Different genotype combinations in the three groups of children; the unvaccinated, partially vaccinated, and fully vaccinated were observed. The combination G1P[8] was the most common in fully vaccinated children. The fact that vaccinated children hospitalized due to RVA infection, with the same genotype from the vaccine, may be related to the high prevalence of chronic malnutrition in this province (55%), the highest in the country [19]. It is known that malnutrition can impair the immune system and weaken the vaccine’s immune response [48, 49]. However, this hypothesis cannot be tested in this analysis due to study design, and we cannot rule out other factors as concomitant enteric infections, histo-blood group antigen, microbiota composition and mothers antibodies [50, 51].

An increased number of strains reported as uncommon such as G3P[4], G9P[4], G9P[6], G2P[6], G12P[8] and non-typeable were observed mainly in fully vaccinated children, highlighting the importance of the continuous surveillance.

The G1P[8] strain was the most common genotype combination in all age groups before the introduction of the vaccine. A high genotype diversity after vaccine introduction was observed in children under 12 months of age. Our findings are not concordant with a study conducted in the southern region of the country in which the diversity of RVA genotypes strains were observed in all age groups during both periods [31]. Also, in Turkey, genotype diversity was common in children from 13 to 24 months of age [52].

Regardless of the vaccine period, RVA was more common in the dry/cold season (April—September) than in the wet/rainy season (October—March), as expected [23, 31].

The study findings should be interpreted carefully as the following limitations were identified i) variables with non-response in the forms, even with continuous training of the staff, ii) unbalanced sample size between pre and post-periods and small sample size to some subgroups, iii) time period in the pre-vaccine was lower than the ideal minimal two years’ period (March—December 2015). However, our sample size meets the minimum sample power (80%) of the main hypothesis tests. This study was carried out at a quaternary-level hospital, which probably included only children with severe disease, where the epidemiological distribution of the disease may be different from hospitals at other levels (tertiary, secondary, rural, post). Despite the limitations, we were able to describe the epidemiology of RVA infection at HCN and the risk factors related to infection in each period (pre- and post-vaccine periods). Some questions need to be addressed, such as: (i) the reason why vaccinated children are infected with RVA to the point of requiring hospitalization; (ii) the role of animals in the occurrence of new/uncommon RVA genotypes to optimize the vaccination.

In conclusion, RVA infection was high in *Hospital Central de Nampula* even after vaccine introduction, mostly in children under 12 months. During the pre-vaccine period, piped water was associated with RVA infection, however, additional studies are needed to investigate this association. In contrast, after vaccine introduction, risk factors for RVA infection were age (0–11 months) and contact with animal (chicken, duck and multiple animals). The infection was more common in vaccinated children, G9P[4] and G1G3P[8] were the most common genotypes in partially vaccinated, while in fully vaccinated G1P[8], G9P[6], and G9P[4] were the most common. Our results show a need for further research to understand the factors behind RVA infection in vaccinated children such as the vaccine immune response and factors that contribute to strains diversity through the whole genome sequence and phylogenetic analysis in Nampula.

## Supporting information

S1 Table Socio-demographic factors related to infection by rotavirus A in the pre and post-vaccine period in children younger than 24 months (N = 499). (DOCX)

S2 Table Genotypes distribution by age group in the pre-vaccine period, N = 60. (DOCX)

S3 Table RVA genotypes distribution by age groups in the post-vaccine period, N = 92. (DOCX)

S1 Dataset Minimal dataset. (XLSX)
