# Supplementary material for: Rotavirus A infection in pre- and post-vaccine period: Risk factors, genotypes distribution by vaccination status and age of children in Nampula Province, Northern Mozambique (2015-2019)
Source: PLoS One. 2021 Aug 6;16(8):e0255720. doi: 10.1371/journal.pone.0255720 (PMC8345880; doi:10.1371/journal.pone.0255720)
Supplement: S1 Table — (DOCX) [file pone.0255720.s001.docx]

**Supporting information**

**S1 Table. Socio-demographic factors related to infection by rotavirus A in the pre and post-vaccine period in children younger than 24 months (N = 499).**

| Characteristics | Pre-vaccine | Column1 | p | Post-vaccine | Column2 | p |
| --- | --- | --- | --- | --- | --- | --- |
|  | n/N | % |  | n/N | % |  |
| **Age categorized (in months)** |  |  | 0.453 |  |  | **0.028** |
| 0-11 | 36/83 | 43.4 |  | 66/217 | 30.4 |  |
| 12-23 | 25/67 | 37.3 |  | 26/132 | 19.7 |  |
| **Sex** |  |  | 0.054 |  |  | 0.763 |
| Male | 28/83 | 33.7 |  | 56/217 | 25.8 |  |
| Female | 33/67 | 49.3 |  | 36/132 | 27.3 |  |
| **Exclusive breastfeed** |  |  | 0.434 |  |  | 0.488 |
| Yes | 31/70 | 44.3 |  | 14/61 | 23.0 |  |
| No | 30/79 | 38.0 |  | 78/286 | 27.3 |  |
| **Animal contact** |  |  | 0.876 |  |  | **0.001** |
| No | 50/123 | 40.7 |  | 17/111 | 15.3 |  |
| Yes | 11/26 | 42.3 |  | 75/238 | 31.5 |  |
| **Source of drinking water** |  |  |  |  |  |  |
| **Public tap** |  |  | 0.960 |  |  | 0.621 |
| No | 43/106 | 40.6 |  | 31/125 | 24.8 |  |
| Yes | 16/39 | 41.0 |  | 61/224 | 27.2 |  |
| **Piped water** |  |  | 0.001 |  |  | 0.917 |
| No | 29/95 | 30.5 |  | 74/282 | 26.2 |  |
| Yes | 30/50 | 60.0 |  | 18/67 | 26.9 |  |
| **Borehole/well** |  |  | 0.001 |  |  | 0.405 |
| No | 46/90 | 51.1 |  | 80/294 | 27.2 |  |
| Yes | 13/55 | 23.6 |  | 12/55 | 21.8 |  |
| **River/lake/lagoon** |  |  | 0.593 ^a^ |  |  | 0.542 ^a^ |
| No | 59/144 | 41.0 |  | 92/347 | 41.0 |  |
| Yes | 0/1 | 0.0 |  | 0/2 | 0.0 |  |
| **Purchased/bottled water** |  |  | NA |  |  | 0.264 ^a^ |
| No | 59/145 | 40.7 |  | 91/348 | 26.1 |  |
| Yes | 0/0 | 0.0 |  | 1/1 | 100.0 |  |
| **Age weaning in months (categorized)** |  |  | 0.078 ^a^ |  |  | 0.153 ^a^ |
| 0-6 | 1/4 | 25.0 |  | 3/10 | 30.0 |  |
| 7-12 | 2/17 | 11.8 |  | 4/35 | 23.1 |  |
| **HIV** |  |  | 1.000 ^a^ |  |  | 1.000 ^a^ |
| Negative | 49/125 | 39.2 |  | 30/153 | 19.6 |  |
| Positive | 3/7 | 42.9 |  | 2/13 | 15.4 |  |
| **Underweight** |  |  | **0.010** |  |  | 0.134 |
| No | 40/84 | 47.6 |  | 64/224 | 28.6 |  |
| Yes | 10/42 | 23.8 |  | 23/110 | 20.9 |  |
| ^a^ Fisher’s Exact Test.  NA: Not applicable  Rotavirus A frequency in the pre-vaccine period is 40.7% (61/150) and in the post-vaccine period is 26.4% (92/349). Sample power is 88.0% | | | | | | |
