# Supplementary material for: Rotavirus A infection in pre- and post-vaccine period: Risk factors, genotypes distribution by vaccination status and age of children in Nampula Province, Northern Mozambique (2015-2019)
Source: PLoS One. 2021 Aug 6;16(8):e0255720. doi: 10.1371/journal.pone.0255720 (PMC8345880; doi:10.1371/journal.pone.0255720)
Supplement: S2 Table — (DOCX) [file pone.0255720.s002.docx]

**Supporting information**

**S2 Table. RVA genotypes distribution by age group in the pre-vaccine period; N=60.**

| Genotypes | Age in months (categorized) | | | | | |
| --- | --- | --- | --- | --- | --- | --- |
|  | **0 - 11** | **%** | **12 - 23** | **%** | **24 - 59** | **%** |
| G1P[6] | 1 | 3.0 | 0 | 0.0 | 0 | 0.0 |
| G1P[8] | 23 | **69.7** | 22 | **100.0** | 2 | **40.0** |
| G2P[6] | 4 | 12.1 | 0 | 0.0 | 2 | **40.0** |
| G9P[8] | 0 | 0.0 | 0 | 0.0 | 1 | 20.0 |
| GX ^b^P[8] | 4 | 12.1 | 0 | 0.0 | 0 | 0.0 |
| GX ^b^P[X] ^a^ | 1 | 3.0 | 0 | 0.0 | 0 | 0.0 |
| Total | 33 | 100.0 | 22 | 100.0 | 5 | 100.0 |
| ^a^ Refers to strains that were non-typeable for P  ^b^ Refers to strains that were non-typeable for G | | | | | | |
