# Supplementary material for: Rotavirus A infection in pre- and post-vaccine period: Risk factors, genotypes distribution by vaccination status and age of children in Nampula Province, Northern Mozambique (2015-2019)
Source: PLoS One. 2021 Aug 6;16(8):e0255720. doi: 10.1371/journal.pone.0255720 (PMC8345880; doi:10.1371/journal.pone.0255720)
Supplement: S3 Table — (DOCX) [file pone.0255720.s003.docx]

**Supporting information**

**S3 Table.** RVA genotypes distribution by age groups in the post-vaccine period; N = 92.

| Genotypes | Age in months (categorized) | | | |
| --- | --- | --- | --- | --- |
|  | **0 - 11** | **%** | **12 - 23** | **%** |
| G1P[8] | 18 | **27.3** | 8 | **30.8** |
| G2P[6] | 3 | 4.5 | 1 | 3.8 |
| G3P[4] | 5 | 7.6 | 1 | 3.8 |
| G3P[8] | 1 | 1.5 | 1 | 3.8 |
| G9P[4] | 16 | 24.2 | 4 | 15.4 |
| G9P[6] | 11 | 16.7 | 5 | 19.2 |
| G9P[8] | 1 | 1.5 | 0 | 0.0 |
| GX ^b^P[4] | 5 | 7.6 | 0 | 0.0 |
| GX ^b^P[6] | 0 | 0.0 | 2 | 7.7 |
| GX ^b^P[8] | 0 | 0.0 | 1 | 3.8 |
| G9P[X] ^a^ | 1 | 1.5 | 0 | 0.0 |
| GX ^b^P[X] ^a^ | 3 | 4.5 | 0 | 0.0 |
| G12P[8] | 1 | 1.5 | 0 | 0.0 |
| G1G3P[8] | 1 | 1.5 | 3 | 11.5 |
| Total | 66 | 100.0 | 26 | 100.0 |
